# COVID‐19 infodemic about nucleic acid amplification tests in Japan

**DOI:** 10.1002/jgf2.504

**Published:** 2021-10-27

**Authors:** Kazuki Shimizu, Kenji Shibuya, Yasuharu Tokuda

**Affiliations:** ^1^ Tokyo Foundation for Policy Research Tokyo Japan; ^2^ Department of Health Policy London School of Economics and Political Science London UK; ^3^ Faculty of Public Health and Policy London School of Hygiene and Tropical Medicine London UK; ^4^ Soma City COVID Vaccination Medical Center Fukushima Japan; ^5^ Muribushi Okinawa Center for Teaching Hospitals Okinawa Japan

The issues on vaccine‐associated mis/disinformation during the COVID‐19 pandemic have highlighted the importance of resolving our current infodemic. In Japan, a lack of capacity in emergency risk communication was already identified as a major issue prior to the current pandemic.^1^ This had resulted in another challenge in early to mid‐2020: an infodemic regarding nucleic acid amplification tests.

The governmental COVID‐19 experts proactively employed a so‐called “cluster‐based” approach in February 2020 to suppress COVID‐19 transmission by mainly focusing on clusters of major symptomatic cases and their close contacts.^2^ While to what extent this approach helped contain and mitigate COVID‐19 transmission has not yet been evaluated, there was a major concern with this approach in terms of lack of scientifically solid evidence and the presence of asymptomatic infections and airborne transmission. Furthermore, despite reservations of its approach in medical professionalism and humanitarianism, as managing COVID‐19 clusters was highly prioritized over caring sporadic cases, government officials and their scientific advisers became complacent about “Japan model” after mitigating the impact of the first wave of COVID‐19 in May 2020,^3^ and they did not assess what worked well and what did not, nor quickly modify and update the strategy to ensure citizens' access to testing. The initial challenges in specimen collection, handling, and transport as well as insufficient testing capacity were never openly reflected upon—a sharp contrast from what was observed in other East Asian and Pacific countries where widespread testing was implemented to control the transmission.^4^

In addition, the official view of the governmental subcommittee on COVID‐19 measures, the successor of expert meeting, was not based on sound science. Despite the principle of nucleic acid amplification tests, and real‐world data from countries that successfully eliminated community transmission of COVID‐19,^5^, ^6^ they employed the specificity of testing as low as 99% or 99.9% without presenting solid empirical evidence to back up these claims^7^, ^8^ and emphasized the issues of false‐positive cases which would potentially bring unnecessary admissions and generate new transmissions. Moreover, the independent investigation commission on the Japanese government's response to COVID‐19, launched by the Asia Pacific Initiative in July 2020, revealed that the government generated and circulated misinformation about COVID‐19 testing. The government's document explained that widespread PCR testing could lead to a significant number of false‐positive cases, overwhelm the overstretched health system capacity, and bring public mistrust toward testing.^9^ On the contrary, health system capacity in Japan was overwhelmed by nosocomial infections because of insufficient testing and a depleted healthcare workforce.^2^

While some Japanese healthcare professionals raised their objections, others remained silent or propagated misinformation during the dispute over testing through multiple media including the social network service. The need to prioritize “deference to political authority”^10^ at the expense of respect for science might have impeded efforts to combat the misinformation generated by the government and its advisors. Additionally, some journalism covering the fields of science and health communication was contradictory to their ethics and standards in truthfulness, accuracy, independence, impartiality, and accountability. They primarily cited governmental views and incited unscientific propaganda to downgrade the gold standard test without showing compelling evidence. While testing could be utilized as one of the tools for reducing individual and societal risks of COVID‐19, and maintaining and promoting behavioral changes, they even argue that expanded testing would create false relief, which assisted in spreading the misinformation, indirectly jeopardized public trust, and might pose a psychological barrier to public access to testing.

During the past decade, challenges in health communication have repeatedly occurred in Japan, such as the misinformation after the Fukushima disaster^11^ and the overall low confidence level toward vaccines.^12^ The COVID‐19 pandemic not only exposed these long‐standing challenges, but explicitly suggested that the government and some healthcare professionals might have also played a central role in promulgating misinformation and may be a driver of the infodemic in Japan.

In conclusion, the COVID‐19 infodemic about nucleic acid amplification tests in Japan resulted from a series of issues embedded in the country's sociopolitical and structural systems, warranting immediate reform. To improve pandemic response efficiency and public health outcomes, Japanese scientists need to adopt a greater level of independence from political and governmental pressures. Revamping scientific journalism and training systems for healthcare professionals to emphasize the importance of their positions in both interprofessional and trans‐professional contexts will be pivotal to enhancing their professional autonomy based on principles of medical ethics. There is a pressing need to establish an effective context‐tailored, evidence‐based risk communication system that manages the infodemic and misinformation to achieve effective health communication during and after the COVID‐19 pandemic. As misinformation tends to be fueled through the internet and social media, conducting network analyses and real‐time social listening will be critical to detecting the nature of these issues and enacting timely interventions. Finally, licensed healthcare professionals must be aware that they possess professional and ethical responsibilities and must act as trusted messengers of public health information. Spreading misinformation and disinformation completely contradicts their professionalism and damages public trust toward all healthcare workers, leaving the nation prone to emerging infectious diseases and chemical, biological, radiological, nuclear (CBRN) threats, and threatening the health of patients and the public.

## CONFLICT OF INTEREST

The authors have stated explicitly that there are no conflicts of interest in connection with this article.
